# Rahmenbedingungen informierter Entscheidungen und interdisziplinärer Zusammenarbeit in der Versorgung von Schwangerschaftsabbrüchen – Perspektiven von Beratungsfachkräften

**DOI:** 10.1007/s00103-026-04209-7

**Published:** 2026-02-26

**Authors:** Maika Böhm, Judit Baer, Eva Kubitza, Petra Brzank, Ulrike Busch, Daphne Hahn, Cornelia Helfferich, Christine Knaevelsrud, Tilman Knittel, Silvia Krumm, Sarah Schumacher

**Affiliations:** 1 Fachbereich Soziale Arbeit. Medien. Kultur, Hochschule Merseburg, Eberhard-Leibnitz-Str. 2, 06217 Merseburg, Deutschland; 2 Fachbereich Sozialmedizin, Rehabilitationswissenschaften und Versorgungsforschung, Hochschule Nordhausen, Nordhausen, Deutschland; 3 Institut für Angewandte Sexualwissenschaft (IfaS), Hochschule Merseburg, Merseburg, Deutschland; 4 Fachbereich Gesundheitswissenschaften, Hochschule Fulda, Fulda, Deutschland; 5 Cornelia Helfferich Institut für Geschlechter- und Familienforschung, Freiburg, Deutschland; 6 Fachbereich Erziehungswissenschaft und Psychologie, Freie Universität Berlin, Berlin, Deutschland; 7 Klinik und Poliklinik für Psychiatrie und Psychotherapie, Universität Leipzig, Leipzig, Deutschland; 8 Institute for Mental Health and Behavioral Medicine, HMU Health and Medical University, Potsdam, Deutschland

**Keywords:** Bedarfsgerechte Versorgung, Qualitative Interviewstudie, Ungewollte Schwangerschaft, Zugangsbarrieren, Schwangerschaftsberatungsstellen, Needs-based care, Pregnancy counselling centres, Qualitative interview study, Unwanted pregnancy, Barriers to access

## Abstract

**Hintergrund:**

Informierte und selbstbestimmte Entscheidungen gelten als zentrales Element menschenrechtskonformer Versorgung von Schwangerschaftsabbrüchen. Der Beitrag untersucht Rahmenbedingungen informierter, selbstbestimmter Entscheidungen und interdisziplinärer Zusammenarbeit aus Sicht von Beratenden aus Schwangerschaftsberatungsstellen in Deutschland. Grundlage ist eine qualitative Studie des Teilprojekts zur psychosozialen Versorgung des Verbundvorhabens „Erfahrungen und Lebenslagen ungewollt Schwangerer – Angebote der Beratung und Versorgung“ (ELSA).

**Methoden:**

Im 4. Quartal 2022 wurden 6 leitfadengestützte Fokusgruppeninterviews mit Fachkräften an 4 Standorten durchgeführt und mittels inhaltlich strukturierender Analyse ausgewertet.

**Ergebnisse:**

Informierte Entscheidungen werden als relationaler Prozess verstanden, der über Wissensvermittlung hinaus Orientierung bietet. Informationsbarrieren, fehlende Sprachmittlung und soziale Ungleichheiten erschweren den Prozess, insbesondere für migrantische und mehrfach belastete Personen. In der interdisziplinären Zusammenarbeit werden Asymmetrien zwischen psychosozialer und medizinischer Versorgung sichtbar. Kooperationen sind häufig personenabhängig, strukturell wenig abgesichert und ausbaufähig.

**Diskussion:**

Informierte und selbstbestimmte Entscheidungen können durch verbesserte interdisziplinäre Vernetzung, barrierearme und verlässliche Informationsangebote sowie institutionalisierte Sprachmittlung unterstützt werden. Der Abbau struktureller Ungleichheiten und die Normalisierung des Schwangerschaftsabbruchs werden als zentral beschrieben und die strafrechtliche Rahmung sowie Pflichtberatung teils kritisch reflektiert. Schwangerschaftsberatungsstellen übernehmen eine zentrale Rolle in der bedarfsgerechten Unterstützung.

## Abstract (en)

**Background:**

Informed and self-determined decisions are a central element of human-rights-compliant abortion care. This article examines the framework conditions for informed, self-determined decisions and interdisciplinary cooperation from the perspectives of counsellors from pregnancy counselling centres in Germany. The data are based on a qualitative study conducted as part of a sub-project on psychosocial care within the joint project “Experiences and life situations of women with unwanted pregnancies—counselling and care services”.

**Methods:**

In the fourth quarter of 2022, six guided focus group interviews were conducted with 25 professionals at four locations and analysed using content-structuring analysis.

**Results:**

Informed decisions are understood as a relational process that offers guidance beyond the transfer of knowledge. Information barriers, a lack of language mediation and social inequalities complicate the process, especially for migrants and people facing multiple challenges. Interdisciplinary collaboration reveals asymmetries between psychosocial and medical care. Collaborations are often dependent on individuals, structurally insecure and in need of improvement.

**Discussion:**

Informed and self-determined decisions can be supported by improved interdisciplinary networking, accessible and reliable information services, and institutionalised language mediation. The reduction of structural inequalities and the normalisation of abortion are described as central issues, and the criminal law framework and compulsory counselling are reflected upon critically in some cases. Pregnancy counselling centres play a key role in this process.

## Hintergrund

Als zentrales Element einer menschenrechtskonformen Versorgung von Schwangerschaftsabbrüchen wird in der 2022 veröffentlichten „Abortion Care Guideline“ der Weltgesundheitsorganisation (WHO; [1]) die Ermöglichung einer informierten und selbstbestimmten Entscheidung benannt. Auch die S2k-Leitlinie „Schwangerschaftsabbruch im ersten Trimenon“ der Arbeitsgemeinschaft der Wissenschaftlichen Medizinischen Fachgesellschaften (AWMF) aus demselben Jahr betont die Relevanz einer informierten Entscheidung [2].

Der Begriff der *informierten Entscheidung* ist eng mit dem Patientenrechtegesetz (2013) verknüpft (§§ 630c–630e Bürgerliches Gesetzbuch [BGB]). Ziel ist es, Patient*innen eine selbstbestimmte Entscheidung auf Grundlage verständlicher, evidenzbasierter Informationen zu Zweck, Durchführung, Erfolgsaussichten sowie Risiken und Folgen medizinischer Maßnahmen zu ermöglichen. Im Kontext von Schwangerschaftsabbrüchen bedarf es einer angepassten Konzeptualisierung der informierten Entscheidung, da es sich nicht um therapeutische Entscheidungen im engeren Sinne handelt. Zudem beziehen sich die für eine informierte Entscheidung notwendigen Informationen nicht nur auf medizinische Aspekte wie Methoden, Ablauf und mögliche Komplikationen, sondern auch auf rechtliche Rahmenbedingungen, Fristen und Zugangswege zur Versorgung. Folgende Definition behält dennoch Gültigkeit: „Als informiert gilt eine Entscheidung, die auf der Basis von ausreichendem Wissen über Vor- und Nachteile im Einklang mit den persönlichen Präferenzen und Überzeugungen getroffen wird“ [3].

Die informierte Entscheidung ist ein zentraler Baustein des Rechts auf *reproduktive Selbstbestimmung*, das international etwa in der UN-Frauenrechtskonvention (CEDAW) und in Deutschland verfassungsrechtlich als Teil des Persönlichkeitsrechts (Art. 2 Abs. 1 i. V. m. Art. 1 Abs. 1 GG) festgehalten ist. Sie bezeichnet das Menschenrecht, „über die Belange des eigenen Lebens und des eigenen Körpers, insofern sie die Fortpflanzung betreffen, selbst zu entscheiden“ [4]. Der Begriff der Selbstbestimmung ist jedoch nicht unumstritten: So wird aus feministischer Perspektive kritisiert, dass ökonomische Abhängigkeiten und weitere strukturelle Ungleichheiten unzureichend mitgedacht würden [5]. In den 1990er-Jahren wurde daher das umfassendere Prinzip *reproduktiver Gerechtigkeit* formuliert, das reproduktive Rechte und soziale Gerechtigkeit eng miteinander verknüpft und „nach den Bedingungen autonomer Entscheidungen, nach diskriminierenden Strukturen, finanziellen Herausforderungen, tatsächlichen Lebensumständen“ fragt [6]. Mit diesem Ansatz wird auch der Zugang zu einer personenzentrierten, diskriminierungssensiblen und qualitativ gesicherten Versorgung bei Schwangerschaftsabbrüchen als integraler Bestandteil reproduktiver Gesundheit verstanden. Er umfasst neben medizinischer und psychosozialer Betreuung den Abbau struktureller Ungleichheiten und den Schutz vor Stigmatisierung.

Im Kontext eines Schwangerschaftsabbruchs bedeutet dies, dass eine informierte und daran anschließend auch selbstbestimmte Entscheidung nur unter Bedingungen möglich ist, die den Zugang zu neutralen, verständlichen Informationen, ausreichend Zeit und nicht bevormundender Unterstützung gewährleisten [1]. Auf Grundlage des gesetzlichen Auftrags aus dem § 219 Strafgesetzbuch (StGB) und dem Schwangerschaftskonfliktgesetz (SchKG) nehmen Schwangerschaftsberatungsstellen dabei eine zentrale Schnittstellenfunktion zwischen individueller Lebenssituation, rechtlichem Rahmen und medizinischer Versorgung ein.

Nachfolgend werden Praxiserfahrungen und Einschätzungen von Beratenden aus Schwangerschaftsberatungsstellen zu den strukturellen, rechtlichen und institutionellen Bedingungen fokussiert, die informierte und selbstbestimmte Entscheidungen im Kontext ungewollter Schwangerschaften und Schwangerschaftsabbrüche in Deutschland rahmen. Es werden zunächst rechtliche Rahmenbedingungen und der Forschungsstand skizziert, anschließend die methodische Vorgehensweise sowie die zentralen Ergebnisse dieser Erhebung vorgestellt.

### Rechtliche Rahmenbedingungen des Schwangerschaftsabbruchs in Deutschland

Schwangerschaftsabbrüche sind in Deutschland im Strafgesetzbuch geregelt (§ 218 StGB). Nach § 218a und § 219 StGB ist ein zwar rechtswidriger, aber straffreier Schwangerschaftsabbruch bis zur 12. Woche nach Empfängnis möglich, wenn die Schwangere eine Schwangerschaftskonfliktberatung bei einer nach § 3 SchKG anerkannten Schwangerschaftsberatungsstelle in Anspruch genommen, den Beratungsschein nach § 7 SchKG erhalten hat und zwischen Beratung und Abbruch mindestens 3 Tage liegen.^1^,^2^ Im Jahr 2024 wurden 96 % aller Abbrüche (2024: 106.455) nach der sog. Beratungsregelung innerhalb der ersten 12 Schwangerschaftswochen durchgeführt [7].

Hinsichtlich der Regulierung von Schwangerschaftsabbrüchen, 3‑tägiger Wartezeit und verpflichtender Beratung gibt es nach wie vor fachliche, juristische, politische und zivilgesellschaftliche Diskussionen. Neuere Entwicklungen sind die Abschaffung des § 219a StGB (2022) und das Verbot von „Gehsteigbelästigungen“ (2024), d. h. Beeinflussungsversuchen vor Beratungsstellen oder medizinischen Einrichtungen. Die von der Bundesregierung einberufene unabhängige „Kommission zur reproduktiven Selbstbestimmung und Fortpflanzungsmedizin“ (2024) empfahl zudem, Abbrüche in der Frühphase der Schwangerschaft außerhalb des StGB zu regeln [4]. Bis zum vorzeitigen Ende der Legislaturperiode in 2025 wurde jedoch keine gesetzliche Änderung beschlossen. Die skizzierten rechtlichen und politischen Entwicklungen bilden den Rahmen, vor dem bisherige empirische Befunde zur psychosozialen und medizinischen Versorgung bei ungewollten Schwangerschaften und Schwangerschaftsabbrüchen einzuordnen sind.

### Empirische Befunde zur psychosozialen und medizinischen Versorgung

Der Zugang zu Versorgung im Kontext von Schwangerschaftsabbrüchen ist ein zentraler Bestandteil reproduktiver Gesundheit. Psychosoziale Versorgung umfasst Informations‑, Unterstützungs- und Begleitangebote vor, während und nach einem Schwangerschaftsabbruch und wird in Deutschland vorrangig durch knapp 1400 gesetzlich anerkannte Schwangerschaftsberatungsstellen umgesetzt [8]. Medizinische Versorgung beinhaltet neben der Feststellung der Schwangerschaft und Schwangerschaftswoche die Aufklärung, Durchführung und Nachsorge des Eingriffs und erfolgt je nach Abbruchmethode und regionaler Versorgungsstruktur in gynäkologischen Praxen, Tageskliniken oder (seltener) in Krankenhäusern [9, 10].

Während die Versorgungslage und strukturellen Rahmenbedingungen im Kontext von Schwangerschaftsabbrüchen in Deutschland lange Zeit nur randständig erforscht wurden, befassen sich jüngere Studien zunehmend mit den Erfahrungen ungewollt Schwangerer sowie den professionellen Einschätzungen medizinischer und psychosozialer Fachkräfte. So liegen mit der ELSA-Studie (Erfahrungen und Lebenslagen ungewollt Schwangerer) erstmals bundesweit erhobene Daten zur Versorgungslage, Inanspruchnahme und Bedarfspassung von Unterstützung aus der Perspektive ungewollt Schwangerer und von Fachkräften vor. Die Ergebnisse weisen auf vielfältige Zugangsbarrieren hin, darunter Informationsdefizite, regionale Versorgungsunterschiede, organisatorische Hürden und stigmatisierende Rahmenbedingungen [9]. Auch Herausforderungen in der interdisziplinären Zusammenarbeit werden sichtbar, insbesondere zwischen Beratungsstellen und medizinischer Versorgung [11]. Zu ähnlichen Befunden kommen auch weitere Studien: Die in der CarePreg-Studie befragten Fachkräfte erachten unterschiedliche Aspekte einer personenzentrierten Versorgung als sehr relevant, aber nicht umfänglich umgesetzt [12]. Auch die in einer Studie von Kolandt et al. befragten Fachkräfte aus der medizinischen und psychosozialen Versorgung beschreiben Informationsbarrieren, Hürden infolge der aktuellen rechtlichen Regelungen sowie gesellschaftliche Stigmatisierung [13]. Eine qualitative Befragung von Schwangerschaftsberater*innen aus Berlin und Brandenburg stellt ebenfalls Hürden auf struktureller und individueller Ebene sowie Unsicherheiten seitens der Fachkräfte fest [14].

Zu den gesetzlichen Vorgaben, unter denen Versorgung und Entscheidungsprozesse im Kontext eines Schwangerschaftsabbruchs stattfinden, zählt auch die Schwangerschaftskonfliktberatung^3^. Die gesetzliche Vorgabe einer auf den „Schutz des ungeborenen Lebens“ abzielenden, aber gleichzeitig ergebnisoffenen Beratung (§ 219 StGB, § 5 SchKG) sowie die strafbewehrte Verpflichtung zur Beratung, die schon im Vorfeld ansetzt, bevor ein negativ sanktioniertes Ereignis eintritt [16], unterscheidet diese Beratung von anderen psychosozialen Beratungsangeboten. Eine klient*innenzentrierte Beratung in diesem Spannungsfeld wird von vielen Beratungsfachkräften als widersprüchlich und verunsichernd für Klient*innen empfunden [17, 18]. Bomert et al. kommen zum Ergebnis, dass Machtasymmetrien vor diesem rechtlichen und institutionellen Hintergrund zwangsläufig bestehen, selbst wenn Fachkräfte den Klient*innen Handlungsspielräume ermöglichen [19].

Studienübergreifend zeigen sich eingeschränkte Informationszugänge, regionale und institutionelle Versorgungsunterschiede, rechtliche Unsicherheiten sowie gesellschaftliche Stigmatisierung als wiederkehrende Problemkonstellationen, die sowohl die Erfahrungen ungewollt Schwangerer als auch die Handlungsspielräume der beteiligten Fachkräfte rahmen. Vor diesem Hintergrund fokussiert die vorliegende Untersuchung die Perspektiven von Beratenden auf strukturelle, rechtliche und institutionelle Einflussfaktoren informierter und selbstbestimmter Entscheidungen im Kontext eines Schwangerschaftsabbruchs. Da Entscheidungsprozesse nicht isoliert, sondern eingebettet in institutionelle Versorgungsstrukturen stattfinden, wird die Zusammenarbeit zwischen Beratungsstellen und medizinischen Versorgungseinrichtungen als zentraler Bestandteil dieser Rahmenbedingungen einbezogen. Daraus ergeben sich folgende Forschungsfragen:

F1: Wie nehmen Beratende die strukturellen, rechtlichen und institutionellen Rahmenbedingungen wahr, die informierte und selbstbestimmte Entscheidungen im Kontext eines Schwangerschaftsabbruchs prägen?

F2: Wie erleben Beratende die Zusammenarbeit zwischen Beratungsstellen und medizinischen Versorgungseinrichtungen im Hinblick auf eine bedarfsgerechte Unterstützung ungewollt Schwangerer?

## Methoden

An der ELSA-Studie war ein Verbund aus 6 Forschungszentren beteiligt, die jeweils eigenständige Teilprojekte verantworteten. Ziel der Studie war es, Erkenntnisse über Belastungen sowie vorhandene Ressourcen von ungewollt Schwangeren zu gewinnen. Darüber hinaus wurde sowohl die medizinische als auch die psychosoziale Angebotsstruktur untersucht. Die Hochschule Merseburg war mit einem Teilprojekt zur psychosozialen Versorgung ungewollt Schwangerer (ELSA-PV) beteiligt, das mehrere Teilerhebungen umfasste:

- die Erhebung von Strukturdaten zur Beratungsstellenlandschaft anhand einer quantitativen Befragung von Leitungskräften,
- qualitative und quantitative Medieninhaltsanalysen zu schwangerschaftsabbruchbezogenen Online-Informationen
- sowie eine qualitative Interviewstudie mit Beratenden zu ihrer Wahrnehmung der psychosozialen und medizinischen Versorgung im Kontext ungewollter Schwangerschaft und Schwangerschaftsabbruch.

Letztere Teilstudie bildet die Datengrundlage für diese Publikation.

Im 4. Quartal 2022 wurden deutschlandweit 6 leitfadengestützte Fokusgruppeninterviews [20] mit insgesamt 25 Schwangerschafts(konflikt)berater*innen an 4 Standorten durchgeführt. Fokusgruppen wurden eingesetzt, um die Praxiserfahrungen und Einschätzungen von Beratenden nicht isoliert, sondern im Austausch mit Kolleg*innen zu erheben. So lassen sich vergleichbare Problematisierungen sowie divergierende Sichtweisen auf strukturelle, rechtliche und institutionelle Rahmenbedingungen sichtbar machen. Die Rekrutierung erfolgte entlang regionaler Cluster und der räumlichen Nähe zu Forschungsstandorten des ELSA-Verbunds (Merseburg, Frankfurt a. M., Hamburg und Ulm). Um diese Standorte wurde ein Radius von 100 km festgelegt, in dem alle nach § 3 SchKG anerkannten Beratungsstellen angeschrieben und zu den Fokusgruppeninterviews eingeladen wurden. Als Incentive für eine Teilnahme wurde ein Wunschgutschein in Höhe von 30 € angeboten. Bei der Stichprobenbildung wurden Trägerzugehörigkeit, konfessionelle Bindung und Beratungsscheinausstellung, die regionale Verteilung innerhalb Deutschlands sowie die Verteilung auf Groß‑, Mittel- und Kleinstädte berücksichtigt (Tab. 1).

**Tab. 1:** Regionale Verteilung der Befragten und die Verteilung der Beratungsscheinausstellung

Nummer	Region	Anzahl der Befragten	Regionstyp	Beratungsscheinausstellung	
		Pro Standort (*n* = 25)	(Kleinstadt, Mittelstadt, Großstadt)	Ja	Nein
1	Ost	5	1 KS, 2 MS, 2 GS	5	0
2	Nord_1	3	1 MS, 2 GS	2	1
3	Nord_2	4	2 MS, 2 GS	4	0
4	West_1	4	2 MS, 2 GS	3	1
5	West_2	4	1 MS, 3 GS	3	1
6	Süd	5	2 MS, 3 GS	5	0

Die übergreifenden Forschungsfragen wurden mithilfe eines Interviewleitfadens operationalisiert, der auf Grundlage der Projektziele, des theoretischen Hintergrunds sowie erster Vorarbeiten im ELSA-Verbund entwickelt wurde. Zentrale Themen der psychosozialen und medizinischen Versorgung ungewollt Schwangerer wurden in offene, explorative Leitfragen überführt und in 4 thematische Blöcke gegliedert: (1) Begriffsverständnis und Relevanz ungewollter Schwangerschaft im Beratungskontext, (2) Einschätzungen zur psychosozialen und medizinischen Versorgung, (3) Informiertheit und Informationszugänge der Ratsuchenden sowie (4) Veränderungsbedarfe aus Sicht der Praxis.

Die Interviews wurden durch 3 wissenschaftliche Mitarbeitende mithilfe eines teilstrukturierten und zuvor erprobten Leitfadens geführt und dauerten zwischen 01:35 h und 02:10 h (Ø-Länge 01:52 h). Nach der Transkription und Anonymisierung erfolgte eine inhaltsanalytische Auswertung nach Kuckartz und Rädiker [21] mit Unterstützung der Software MAXQDA 2024 (Verbi Software GmbH, Berlin, Deutschland). Die Auswertung kombinierte ein deduktiv-induktives Vorgehen: Deduktive Hauptkategorien wurden theorie- und leitfadenorientiert entwickelt, induktive Kategorien materialnah im offenen Codieren ergänzt und im Analyseprozess präzisiert. Zur Qualitätssicherung wurde ein Pretest durchgeführt, in dem 20 % des Materials von 2 Forschenden unabhängig codiert und anschließend kommunikativ validiert wurden; auf dieser Grundlage codierte eine Forscherin das vollständige Datenmaterial.

## Ergebnisse

### Rahmenbedingungen für informierte, selbstbestimmte Entscheidungen

Die Interviews zeigen, dass rechtliche, medizinische, psychosoziale und gesellschaftliche Bedingungen die Möglichkeiten informierter und selbstbestimmter Entscheidungen bei ungewollter Schwangerschaft wesentlich rahmen. Dabei wird Beratung einerseits als zentraler Ort der Orientierung und Entscheidungsunterstützung beschrieben, andererseits als durch gesetzliche Vorgaben, ärztliche Haltungen und gesellschaftliche Stigmata herausgeforderte Praxis. Im Folgenden werden zunächst die Zugänge zu Informationen und deren Vermittlung, danach rechtliche Rahmenbedingungen, ärztliche Interaktionen sowie schließlich lebensweltliche Faktoren und Entscheidungskontexte beschrieben.

#### Zugänge zu Informationen und Beratung als Raum zur Orientierung

Die Beratenden berichten, dass ungewollt Schwangere erste Informationen zu einem möglichen Schwangerschaftsabbruch meist über das Internet, soziale Medien oder persönliche Kontakte erhalten. Ärzt*innen werden von Klient*innen teilweise als hilfreiche Informationsquelle wahrgenommen, vor allem zu medizinischen Fragen. Einige Mediziner*innen würden jedoch selbst keine ausführlichen Informationen zur Verfügung stellen und lediglich auf das verpflichtende Beratungsgespräch verweisen.

In allen Fokusgruppen werden Sprachbarrieren als eine der zentralen Herausforderungen im Zugang zu verlässlichen Informationen beschrieben. Dabei zeigen sich regionale Unterschiede im Umgang mit Sprachmittlung in medizinischer wie psychosozialer Versorgung: von gut funktionierenden Kooperationen mit geschulten Dolmetscher*innen, die kurzfristig verfügbar und thematisch sensibilisiert sind, bis hin zu Übersetzungen durch Partner oder andere Begleitpersonen. Ein Mangel an institutionalisierten und verlässlichen Strukturen für Sprachmittlung kann sich negativ auf die Informiertheit und Entscheidungssicherheit von ungewollt Schwangeren auswirken und zudem zu zeitlichen Verzögerungen führen.

Informiertheit wird von den Befragten jedoch nicht nur als Sachkenntnis über Abläufe und medizinische Fakten verstanden, sondern als mehrdimensionales Konzept, das auch die Fähigkeit umfasst, Informationen kritisch einzuordnen und in Beziehung zur eigenen Lebenssituation zu setzen. Die Beratung wird dabei als zentraler Ort der Informationsvermittlung und -richtigstellung beschrieben, der Reflexion, Klärung von Unsicherheiten, Bearbeitung emotionaler Belastungen und eine Stärkung der Entscheidungsfähigkeit sowie Selbstbestimmung der Klient*innen ermöglicht.

#### Rechtliche Vorgaben als ambivalenter Rahmen der Beratungspraxis

Die gesetzlich vorgeschriebene Pflichtberatung wird von den Befragten in allen Interviews intensiv diskutiert und ambivalent bewertet: Einerseits sichert sie einen Teil der Finanzierung der Beratungsstellen und damit den Zugang zu psychosozialer Unterstützung. Andererseits werden in allen Gruppeninterviews der strafrechtliche Rahmen und seine normativen Auswirkungen auf ungewollt Schwangere kritisch reflektiert und von vielen Befragten die Pflichtberatung als Herausforderung für das eigene berufliche Handeln skizziert. Die Befragten kritisieren dabei insbesondere den normativen Widerspruch zwischen ergebnisoffener Beratung und dem gesetzlich formulierten „Schutz des ungeborenen Lebens“ im StGB und SchKG. Zugleich wird auf die Wirkung der gesetzlichen Pflichtvorgabe auf ungewollt Schwangere hingewiesen. Gerade diejenigen, die bereits vor dem Beratungsgespräch eine Entscheidung für einen Abbruch getroffen haben, berichten den Beratenden gegenüber davon, die verpflichtende Beratung als formale Hürde zu erleben. Der gesetzliche Rahmen führe oft zu Verunsicherung und dem Gefühl, sich rechtfertigen zu müssen, um die Beratungsbescheinigung und damit Zugang zum Abbruch zu erhalten. In dieser Struktur, so wird betont, liege ein grundlegender Konflikt, der sich bis in die alltägliche Beratungspraxis und die Erfahrung begrenzter Selbstbestimmung der Frauen fortsetzt.

In einigen Fokusgruppen wird der Wunsch geäußert, den bestehenden Pflichtcharakter in ein Recht auf Beratung zu überführen: ein niedrigschwelliges, freiwilliges und thematisch breiteres Angebot, das alle Aspekte sexueller und reproduktiver Gesundheit einschließt. Dazu gehören auch flexible Formate wie Telefon- oder Videoberatung, die sich insbesondere seit der COVID-19-Pandemie etabliert haben.

#### Wahrnehmung ärztlicher Kommunikation und Versorgungspraktiken

Die Befragten berichten von großen Unterschieden in den Aufklärungspraktiken und Haltungen von Mediziner*innen, von denen sie in den Beratungen erfahren oder die sie im persönlichen Austausch erleben. Neben sensiblen und unterstützenden Kontakten schildern sie übergriffige oder bevormundende Kommunikation – etwa das ungefragte Zeigen von Ultraschallbildern oder moralische Kommentare – sowie Verzögerungen medizinischer Termine, trotz Mitteilung eines gewünschten Schwangerschaftsabbruchs. Einige Ärzt*innen würden sich zudem zunehmend auf medizinische Informationsvermittlung konzentrieren und psychosoziale Aspekte oder Informationen zum weiteren Ablauf nicht vermitteln. Das Zurückhalten von Informationen, etwa zur 3‑tägigen Wartezeit zwischen Beratung und Abbruch, kann selbstbestimmte Entscheidungen zusätzlich erschweren. Andere Ärzt*innen, etwa jene, die medikamentöse Abbrüche im Home Use ermöglichen, werden hingegen als Akteur*innen wahrgenommen, die zu mehr Selbstbestimmung und Entlastung beitragen.

In den Schilderungen der Beratenden zeigen sich ambivalente Zuschreibungen ärztlicher Rollen – zwischen Kontrolle über Zugang und Ablauf auf der einen und der Ermöglichung informierter, selbstbestimmter Entscheidungen auf der anderen Seite. Beratung wird dabei von den Befragten als Ort verstanden, an dem medizinische Informationen kontextualisiert und mit psychosozialer Unterstützung verbunden werden.

#### Lebensweltliche Bedingungen und Entscheidungskontexte

Die befragten Fachkräfte beschreiben die Entscheidungsprozesse ungewollt Schwangerer als in komplexe Lebenswelten eingebettete Prozesse. Die ihnen berichteten Gründe erleben sie als multifaktoriell und kontextabhängig: Häufig werden die biografische Passung der Schwangerschaft zur jeweiligen Lebensphase, partnerschaftliche Stabilität sowie Vereinbarkeitsprobleme von Beruf, Familie und ökonomischer Sicherheit thematisiert. Manche Beratende beschreiben, dass Frauen den Abbruch auch als Akt der Verantwortung begreifen – aus Sorge um ihre Kinder oder um die Stabilität ihres familiären Alltags. Finanzielle Aspekte werden zwar regelmäßig erwähnt, jedoch meist ergänzend zu anderen Beweggründen. Einige Fachkräfte vermuten, dass solche Begründungen in der Pflichtberatung teils auch als sozial akzeptierte Rechtfertigungen angeführt werden, um die eigene Entscheidung nachvollziehbar zu machen.

Die Entscheidungsprozesse werden in den Interviews nicht moralisch, sondern lebenspraktisch und relational gerahmt, wodurch der Abbruch als legitime Option innerhalb individueller Lebensverläufe nachvollziehbar wird. Lebensweltliche Bedingungen beeinflussen Entscheidungsfähigkeit, Risikoabwägung und Verantwortungsbewusstsein direkt und sind somit zentral für informierte Entscheidungen.

Die Ergebnisse verdeutlichen, dass informierte und selbstbestimmte Entscheidungen ungewollt Schwangerer durch ein Zusammenspiel struktureller, professioneller und lebensweltlicher Faktoren beeinflusst werden. Eine bedarfsgerechte Unterstützung erfordert daher eine enge Abstimmung zwischen psychosozialer Beratung und medizinischer Versorgung, deren konkrete Ausgestaltung im Folgenden betrachtet wird.

### Bedarfsgerechte interdisziplinäre Unterstützung

Wie diese Zusammenarbeit in der Praxis gelingt, zeigt sich in den Erfahrungen der Beratungsfachkräfte mit bestehenden Kooperationsstrukturen, Schnittstellenproblemen und Defiziten in der medizinischen Versorgung.

#### Erlebte Zusammenarbeit und Schnittstellenprobleme

Die befragten Beratenden schildern die Kooperation zwischen Beratungsstellen und medizinischen Einrichtungen überwiegend als einseitig und durch sie selbst getragen. Nach ihrer Wahrnehmung liegt die Hauptverantwortung für den Aufbau und die Pflege von Kontakten bei den Beratenden: Sie recherchieren aktiv regionale Angebote für Schwangerschaftsabbrüche, suchen den Austausch mit gynäkologischen Praxen, hospitieren – sofern möglich – bei Abbrüchen und stellen im Rahmen ihres gesetzlichen Auftrags medizinische Informationen im Beratungsgespräch bereit.

Ärzt*innen würden, so die Befragten, nicht zuverlässig Kontaktdaten von Beratungsstellen weitergeben; ärztlicherseits initiierte Vernetzungen seien selten. Insgesamt beschreiben die Fachkräfte die bestehenden Informationsflüsse, Verweisungen und Kooperationen als stark vom individuellen Engagement Einzelner abhängig und erleben diese vielfach als von ihrer Initiative geprägt. Sie berichten zudem, dass es besonders stabile Kooperationen mit Ärzt*innen gäbe, die selbst Abbrüche anbieten; diese zeigen sich offener, zugänglicher und kooperativer. Diejenigen, die keine Abbrüche durchführen, werden hingegen häufiger als zurückhaltend oder weniger engagiert erlebt. In der Konsequenz führe das überlastete Gesundheitssystem vielerorts dazu, dass ungewollt Schwangere, abhängig von ihrem Wohnort und ihrer Anbindung an eine*n Gynäkolog*in, aufwendigere Wege zurücklegen müssten.

Die Verantwortung für die Sicherstellung bedarfsgerechter Informationen und Vermittlung liegt damit überwiegend bei den Beratungsstellen, während die Kontinuität der Unterstützung ungewollt Schwangerer von der Initiative einzelner Ärzt*innen abhängt.

#### Wahrgenommene Defizite in der medizinischen Versorgung

Alle interviewten Fachkräfte berichten von einer aktuellen oder bevorstehenden regionalen Unterversorgung mit Abbrucheinrichtungen. Häufig zeigt sich dies in Wartezeiten oder weiten Fahrstrecken, was insbesondere für Frauen ohne eigenes Fahrzeug oder in ländlichen Gebieten die Inanspruchnahme erschwert. Ein wiederkehrendes Thema in den Interviews ist der Generationenwechsel: Viele ältere Gynäkolog*innen würden in Rente gehen, während Nachfolger*innen das Angebot nicht fortführen, sodass bestehende Lücken zunehmen. Auch wird durch mehrere Befragte die begrenzte Methodenwahl kritisiert, insbesondere in Bezug auf einen medikamentösen Abbruch oder einen instrumentellen Eingriff unter Lokalanästhesie. Vereinzelt wird auch berichtet, dass Ärzt*innen die gesetzlich mögliche Frist für einen Abbruch bis zur 12. Schwangerschaftswoche nicht nutzen würden und Abbrüche nur bis zu einem früheren Zeitpunkt anbieten.

Darüber hinaus wird ein gravierender Mangel an psychotherapeutischen Angeboten beschrieben, der vor allem ungewollt Schwangere mit psychischen Vorerkrankungen oder Belastungen trifft. Dies könne zu einer Verlagerung therapeutischer Themen in die Beratung führen, was jedoch die Möglichkeiten der Beratungsstellen sowohl inhaltlich als auch ressourcenbedingt übersteigt.

Insgesamt zeigen die Aussagen, dass die medizinische Versorgung nicht nur quantitativ begrenzt ist, sondern auch durch strukturelle Faktoren und organisatorische Rahmenbedingungen bestimmt wird. Die Fachkräfte betonen, dass die Sicherstellung eines flächendeckenden und methodisch vielfältigen Angebots zentral für eine informierte und selbstbestimmte Entscheidung ungewollt Schwangerer ist.

## Diskussion

Die Ergebnisse der vorliegenden Auswertungen verdeutlichen mit Blick auf die erste Forschungsfrage, dass informierte und selbstbestimmte Entscheidungen im Kontext ungewollter Schwangerschaften maßgeblich durch strukturelle, rechtliche und institutionelle Rahmenbedingungen der Versorgung geprägt werden. Dies schließt an die Abortion Care Guideline der WHO an, die informierte und selbstbestimmte Entscheidungen als Kern menschenrechtskonformer Versorgung begreift und deren Ermöglichung in engem Zusammenhang mit zugänglichen und verständlichen Informationen, zeitlichen Handlungsspielräumen sowie nichtdirektiven und diskriminierungsfreien Beratungs- und Unterstützungsangeboten sieht [1].

Die untersuchten Perspektiven der Beratenden ergänzen und vertiefen Befunde aus vorhandenen Studien [9, 12, 19], in denen Entscheidungsprozesse ungewollt Schwangerer als in soziale, rechtliche und versorgungsbezogene Kontexte eingebettet beschrieben werden. Mit Blick auf die erste Forschungsfrage zeigen die hier präsentierten Ergebnisse, wie diese Rahmenbedingungen aus professioneller Sicht in der Beratungspraxis erlebt bzw. wirksam werden. In Übereinstimmung mit Ergebnissen der CarePreg-Studie [12] wird Informiertheit in den Interviews nicht als bloße Wissensvermittlung, sondern als relationaler Prozess beschrieben, der Orientierung, Einordnung und emotionale Verarbeitung erfordert. Beratung übernimmt hier eine zentrale kompensatorische Funktion angesichts fragmentierter Informationen, normativer Verunsicherung und struktureller Barrieren.

Besonders deutlich wird dies im Umgang mit Sprachbarrieren und fehlenden institutionalisierten Strukturen für Sprachmittlung. Die Befunde verweisen auf erhebliche Zugangsungleichheiten, die informierte und selbstbestimmte Entscheidungen insbesondere für migrantische und mehrfach belastete Personen erschweren. Dies deckt sich auch mit den Befunden von Kolandt et al. [13]. Aus der Perspektive reproduktiver Gerechtigkeit wird somit sichtbar, dass Selbstbestimmung von sozialen und institutionellen Bedingungen abhängt und damit die Möglichkeiten zu ihrer Realisierung nicht für alle ungewollt Schwangeren gleichermaßen gegeben sind.

Auch der rechtliche Rahmen des Schwangerschaftsabbruchs erweist sich als ambivalent, ähnlich wie es frühere Studien zeigen [16–18, 22]. Die Pflichtberatung gewährleistet zwar den Zugang zu psychosozialer Unterstützung, steht jedoch zugleich in einem Spannungsverhältnis zum Anspruch ergebnisoffener Beratung. Diese Ambivalenz spiegelt sich unter anderem in der Wahrnehmung ungewollt Schwangerer wider, die die Beratung teils als formale Hürde erleben. Vergleichbare Befunde berichten auch Kolandt et al., die auf die normativen Effekte der rechtlichen Rahmung und deren Einfluss auf Entscheidungserleben und Selbstbestimmung hinweisen [13]. Der von einigen Beratenden geäußerte Wunsch nach einem Recht auf freiwillige Beratung und allgemein einer Entkriminalisierung lässt sich vor diesem Hintergrund als professionsethische Positionierung zugunsten einer autonomiestärkenden Versorgung interpretieren.

In Bezug auf die zweite Forschungsfrage verdeutlichen die Ergebnisse zur interdisziplinären Zusammenarbeit zudem eine Asymmetrie zwischen psychosozialer Beratung und medizinischer Versorgung. Informationsvermittlung und Vernetzung werden weitgehend von Beratungsstellen getragen, während interdisziplinäre Kooperationen häufig vom Engagement einzelner Akteur*innen abhängen. Mit Blick auf medizinische Versorgungsstrukturen zeigt sich in Übereinstimmung mit ELSA und CarePreg [12], dass regionale Unterversorgung, eingeschränkte Methodenwahl und lange Wege als strukturelle Grenzen selbstbestimmter Entscheidungen gedeutet werden, selbst wenn grundsätzlich Informiertheit gegeben ist. Deutlich wird zudem die Notwendigkeit eines strukturierten Austauschs zwischen Beratungsstellen und ärztlichen Praxen bzw. gynäkologischen Kliniken, aber auch mit Akteuren wie dem BIÖG (Bundesinstitut für Öffentliche Gesundheit), um Informationsflüsse und interdisziplinäre Versorgung zu verbessern. Gemeinsame Fortbildungen, fachübergreifende Reflexionsräume und verbindliche Kommunikationsstrukturen werden beispielhaft als Instrumente benannt, um Kooperation und Qualitätssicherung zu stärken.

Eine bedarfsgerechte Versorgung sollte auf informierte, barrierearme und selbstbestimmte Entscheidungsprozesse ausgerichtet sein, die reproduktive Rechte umfassend respektiert und unterstützt. Nachhaltige Verbesserungen erfordern nicht nur strukturelle Maßnahmen und die Stärkung interdisziplinärer Kooperation, sondern auch eine gesellschaftliche und institutionelle Normalisierung sowie Entstigmatisierung des Schwangerschaftsabbruchs als selbstverständlichen Teil von Versorgung. Eine inklusive, diskriminierungssensible Praxis, die psychische Belastungen, Sprachbarrieren und weitere Vulnerabilitätsfaktoren berücksichtigt, bildet hierfür eine zentrale Voraussetzung. Schwangerschaftsberatungsstellen kommt dabei eine Schlüsselrolle zu: Sie schaffen Schnittstellen zwischen medizinischer, psychosozialer und rechtlicher Versorgung und können wesentlich zur Ermöglichung informierter und selbstbestimmter Entscheidungen beitragen.

### Limitationen

Die Ergebnisse der vorliegenden Interviewstudie sind aufgrund der geringen Stichprobengröße nicht generalisierbar und beruhen ausschließlich auf den Aussagen von Beraterinnen, da keine Teilnehmenden anderer Geschlechter rekrutiert werden konnten. Darüber hinaus sind Beratungsstellen aus ländlichen Regionen sowie in katholischer Trägerschaft, die keine Beratungsbescheinigungen ausstellen, im Sample unterrepräsentiert. In der Verteilung der Beratungsstellen innerhalb der Grundgesamtheit überwiegen ebenfalls Groß- und Mittelstädte. Inwieweit die teilnehmenden Beratungsstellen das ländliche Umland zum Teil mitversorgen, lässt sich nicht systematisch auswerten. Ein zentraler Limitationspunkt besteht darin, dass die Studie ausschließlich Perspektiven von Beratungsfachkräften erfasst. Damit wird eine wichtige Berufsgruppe der psychosozialen Versorgung berücksichtigt, zugleich bleiben jedoch die Einschätzungen weiterer relevanter Akteur*innen wie die der Ärzt*innen oder Schwangeren unberücksichtigt.

## Fazit

Die Ergebnisse zeigen, dass Beratende soziale Ungleichheiten und damit verbundene Hürden ebenso wie regionale Unterschiede in medizinischer Versorgung, ärztlichen Praktiken und Haltungen als zentrale Rahmenbedingungen informierter und selbstbestimmter Entscheidungen bewerten. Die Beratung wird als orientierende Unterstützung verstanden, die hilft, Informationen einzuordnen, Handlungsspielräume zu klären und Entscheidungsprozesse unter diesen Bedingungen zu begleiten. Zugleich wird der rechtliche Rahmen als ambivalent und herausfordernd für das eigene Beratungshandeln, teilweise aber auch für die ungewollt Schwangeren beschrieben. Eine enge, gemeinsam verantwortete interdisziplinäre Zusammenarbeit zwischen psychosozialer Beratung und medizinischer Versorgung wird von den Beratenden als zentrale Voraussetzung einer bedarfsgerechten Versorgung angesehen.

Insgesamt wird deutlich, dass informierte und selbstbestimmte Entscheidungen neben individuellen lebensweltlichen Faktoren wesentlich durch strukturelle und institutionelle Rahmenbedingungen ermöglicht werden. Maßnahmen zur Stärkung der interdisziplinären Vernetzung, zur Verbesserung barrierearmer Informationszugänge und zum Ausbau strukturierter Sprachmittlung sollten sowohl in der Praxis umgesetzt als auch in zukünftiger Forschung systematisch untersucht werden. Für ein umfassenderes Verständnis der Versorgungsrealität und der Einflussfaktoren auf informierte Entscheidungen sind sowohl die Perspektiven Betroffener als auch jene der multiprofessionellen Fachkräfte systematisch einzubeziehen.

## Data Availability

Das genutzte Erhebungsinstrument sowie die während der vorliegenden Studie erzeugten Datensätze sind auf begründete Anfrage bei der Korrespondenzperson erhältlich.

## Footnotes

1 Die Regelungen nach medizinischer und kriminologischer Indikation werden an dieser Stelle ausgeklammert.

2 Ärzt*innen mit einer staatlichen Berechtigung für die Schwangerschaftskonfliktberatung können ebenfalls eine Beratungsbescheinigung ausstellen, jedoch darf die Beratungsbescheinigung nicht von derselben Person ausgestellt werden, die den Schwangerschaftsabbruch vornimmt.

3 Die Verwendung des Begriffs „Schwangerschaftskonflikt“ und „Schwangerschaftskonfliktberatung“ enthält oftmals erhebliche Unschärfen (kritisch dazu bspw. [15]). Er wird nachfolgend nur genutzt, wenn auf entsprechend benannte Gesetzestexte verwiesen wird oder er Teil von Interviewaussagen war.
